# Comparative Activity of Ciprofloxacin, Levofloxacin and Moxifloxacin against *Klebsiella pneumoniae*, *Pseudomonas aeruginosa* and *Stenotrophomonas maltophilia* Assessed by Minimum Inhibitory Concentrations and Time-Kill Studies

**DOI:** 10.1371/journal.pone.0156690

**Published:** 2016-06-03

**Authors:** Antoine Grillon, Frédéric Schramm, Magali Kleinberg, François Jehl

**Affiliations:** Institute of Bacteriology, Faculty of Medicine, University of Strasbourg and Strasbourg University Hospital, Strasbourg, France; University of Pittsburgh, UNITED STATES

## Abstract

The aim of this study was to compare the *in vitro* susceptibility of *Klebsiella pneumoniae*, *Pseudomonas aeruginosa* and *Stenotrophomonas maltophilia* to three fluoroquinolones. The minimum inhibitory concentrations (MICs) to ciprofloxacin, levofloxacin and moxifloxacin were examined by E-test^®^ for a total of 40 *K*. *pneumoniae* strains, 40 *S*. *maltophilia* strains and 40 *P*. *aeruginosa* strains. Then, the bactericidal activity of these fluoroquinolones was investigated on five strains of each bacterial species by means of time-kill curves. For *K*. *pneumoniae* and *P*. *aeruginosa*, the distance of the measured MIC from the clinical break-point is a good indicator of the bactericidal activity for ciprofloxacin and levofloxacin as obtained in our experiments. The lower the MIC, the better the bactericidal activity in term of CFU Log decreases. If MIC of ciprofloxacin and levofloxacin against the considered bacteria are far from clinical breakpoint, these two antibiotics are equivalent. According to our MIC_50_ and modal MIC, the breakpoints of both ciprofloxacin and levofloxacin seem to be somewhat high and data suggest reducing them. On *S*. *maltophilia*, none of the tested antibiotics showed a satisfactory activity.

## Introduction

Gram-negative bacteria such as *Enterobacteriaceae*, *P*. *aeruginosa* and *S*. *maltophilia* are common nososomial pathogens. Among *Enterobacteriaceae*, *Klebsiella* is frequently isolated from hospitalized patients [1]. The Urinary tract is the most common site of infection by this pathogen in immunocompromised patients [2], but other infections such as pneumonia, bacteremia, wound infections, nosocomial infections in intensive care unit or neonatal septicemia are frequent [1]. *P*. *aeruginosa*, in particular, is well recognized opportunist pathogen in immunocompromised patients [3], with a mortality rate as high as 50% [4]. It is the most prevalent pathogen among patients with cystic fibrosis [5], and is more common in adults. *S*. *maltophilia* is an environmental bacterium that can cause respiratory-tract infections in humans [6]: 2% of hospitalized patients in intensive care units (ICU) develop colonization or infection [7], and at least 30% of patients with cystic fibrosis are colonized [8, 9]. Although *S*. *maltophilia* is not a highly virulent pathogen, it can lead to severe infections in immunocompromised patients, with a mortality rate up to 37.5% [10].

Fluoroquinolones are currently among the most heavily prescribed antimicrobials in the world because of their spectrum of activity, their pharmacokinetic profiles, and their generally good tolerance. The older narrow-spectrum ciprofloxacin is usually active against Gram-negative bacteria like *Enterobacteriaceae*, *P*. *aeruginosa* or *S*. *maltophilia*, but anaerobic bacteria and some Gram-positive bacteria like *Enterococcus* spp., *Streptococcus* spp. and *Listeria* spp. are naturally resistant. In the 2000s, new fluoroquinolones –levofloxacin and moxifloxacin– were developed, exhibiting enhanced potencies with very low MICs against Gram-positive organisms and/or anaerobes, while maintaining Gram-negative activity [11–13]. They also demonstrated improved PK profiles, characterized by a better systemic distribution particularly in respiratory tract tissues and fluids [14].

This enhanced PK profiles result in large areas under the serum concentrations versus time curves (AUCs) and high peak concentrations. That, in combination with their low MICs, allows them to achieve optimal PK/PD parameters for both efficacy such as AUC_0-24_/MIC (AUC_0-24_ is the area under the concentration-time curve from 0 to 24 h), or prevention of de novo resistance like C_max_/MIC (C_max_ is the maximum plasma concentration). AUC_0-24_/MIC ratio should be superior to 125: if this ratio is lower than 125, treatment failure increase by three to four, and if it is greater than 250, recovery time is enhanced [15, 16]. The C_max_/MIC ratio must reach at least 10 to 12 in order to prevent emergence of de novo resistant mutants [15, 16].

The purpose of the present work was to assess the respective potency of either ciprofloxacin, levofloxacin or moxifloxacin against *K*. *pneumoniae*, *P*. *aeruginosa* and *S*. *maltophilia* by (i) comparing their MICs against a large set of clinical strains of *K*. *pneumoniae*, *P*. *aeruginosa* and *S*. *maltophilia*; (ii) comparing their bactericidal activities through time-kill curves performed on selected strains of each bacteria.

## Materials and Methods

### Bacterial strains

120 clinical strains were obtain from the archived collection of laboratory of clinical microbiology of the University Hospital of Strasbourg was constituted as follows: 40 strains of *K*. *pneumoniae*, 40 strains of *P*. *aeruginosa* and 40 strains of *S*. *maltophilia*. All strains were stored at -80°C using MAST CRYOBANK (Mast diagnostic, Amiens, France).

### MICs measurements

**Fig 1:**
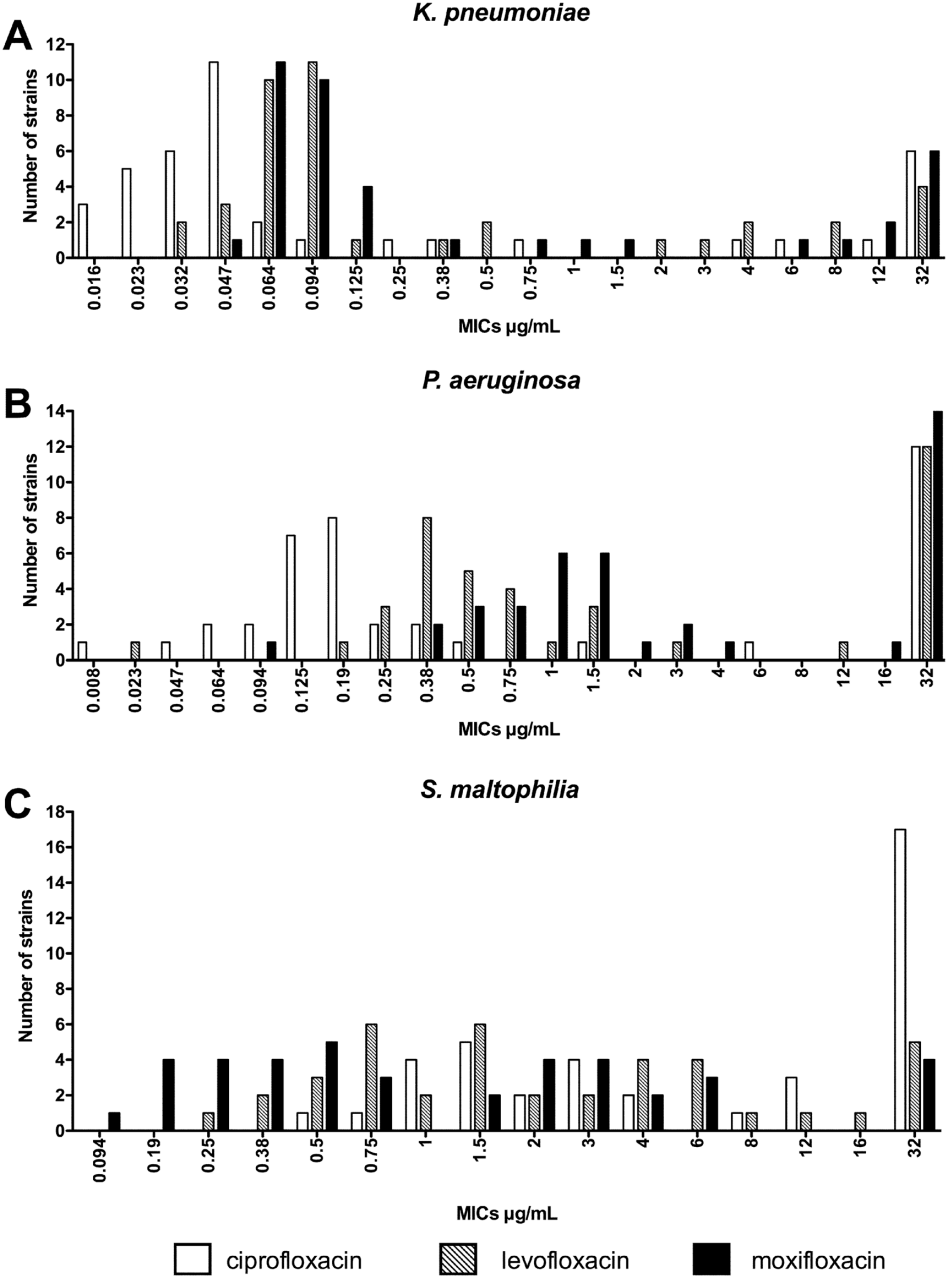
MICs distributions for *K*. *pneumonia* (A), *P*. *aeruginosa* (B), *S*. *maltophilia* (C) against ciprofloxacin, levofloxacin and moxifloxacin.

MICs were determined by the gradient strips method using E-test^®^ (bioMérieux, France) on Mueller-Hinton agar inoculated with a standard inoculum (10^5^ to 10^6^ CFU/ml) according to The European Committee on Antimicrobial Susceptibility Testing (EUCAST) guidelines [17] (Table 1; Fig 1).

**Table 1:** MIC_50_ MIC_90_ and modal MICs for each bacteria and antibiotics.

	MIC_50_	MIC_90_	Modal MIC	EUCAST breakpoints
*K*. *pneumoniae* (n = 40)				
Ciprofloxacin	0.047	>32	0.047	0.5–1
Levofloxacin	0.094	8	0.094	1–2
Moxifloxacin	0.094	>32	0.064	0.5–1
*P*. *aeruginosa* (n = 40)				
Ciprofloxacin	0.19	>32	>32	1–2
Levofloxacin	0.75	>32	>32	1–2
Moxifloxacin	1.5	>32	>32	1–2
*S*. *maltophilia* (n = 40)				
Ciprofloxacin	4	>32	>32	1–2
Levofloxacin	1.5	>32	0.75 and 1.5	1–2
Moxifloxacin	0.75	6	0.5	1–2

MICs and EUCAST breakpoints are expressed in mg/L.

### Time-kill studies

Antibiotics were supplied by their respective manufacturers as pure titrated powders (ciprofloxacin and moxifloxacin by Bayer Healthcare SAS, levofloxacin by Sanofi-Aventis, France). Five strains of each species were selected for time-kill studies, based on their different susceptibility patterns to the antibiotics (Table 2). For each strain, time kill studies were performed for the three molecules at concentrations equal to the theoretical plasma peak (ciprofloxacin = 4 μg/mL; levofloxacin = 10 μg/mL; moxifloxacin = 3 μg/mL), then at concentrations equal to one fold and two fold the MIC of the antibiotic used. Bacteria were initially cultured for 24 h at 37°C on Mueller-Hinton agar. These cultures were then considered as being on stationary growth-phase and used to prepare exponential growth phase at standard inoculum (10^6^ CFU/mL) in Mueller-Hinton broth (MHB, bioMérieux, France). The inoculum of 10^6^ CFU/ml was obtained by standardizing optical density at 550 nm to 0.125 followed by a 1:100 dilution. Final suspensions of bacteria were supplemented with ciprofloxacin, levofloxacin or moxifloxacin at different concentrations and cultured for 24 h at 37°C. Culture aliquots of 100 ml were removed at 2, 4, 6 and 24 h and plated on agar for colony counts.

**Table 2:** MICs (μg/mL) of the bacteria strains tested by time-kill studies.

	Ciprofloxacin		Levofloxacin		Moxifloxacin	
	MICs	Categorization	MICs	Categorization	MICs	Categorization
*K*. *pneumoniae*						
Strain 1	0.016	S	0.032	S	0.064	S
Strain 2	0.75	I	0.5	S	1	I
Strain 3	0.38	S	0.5	S	1.5	R
Strain 4	0.047	S	0.094	S	0.125	S
Strain 5	4	R	4	R	8	R
*P*. *aeruginosa*						
Strain 1	0.125	S	1	S	1	S
Strain 2	0.38	S	1.5	I	4	R
Strain 3	0.008	S	0.023	S	0.094	S
Strain 4	0.5	S	1.5	I	32	R
Strain 5	1.5	I	1.5	I	16	R
*S*. *maltophilia*						
Strain 1	0.5	S	0.25	S	0.094	S
Strain 2	1	S	0.38	S	0.19	S
Strain 3	>32	R	3	R	2	I
Strain 4	3	R	1	S	0.38	S
Strain 5	3	R	1	S	0.25	S

## Results

### Strains MICs

MICs –as determined by E-test^®^ for ciprofloxacin, levofloxacin and moxifloxacin– are presented in Fig 1. For all species and for each antibiotic tested, MIC_50_, MIC_90_ and modal MIC were calculated (Table 1). Clinical breakpoints used for categorization was those provided by EUCAST [17].

Among all *K*. *pneumoniae* strains, 75% were susceptible to ciprofloxacin and levofloxacin, and 67.5% to moxifloxacin. All ciprofloxacin-resistant strains were also resistant to the other quinolones tested (n = 9). The MICs distributions were bimodal for each antibiotic tested (Fig 1A). For each antibiotic, the MIC_50_ and the modal MIC were in the same order of magnitude, but the MIC_90_ was at least four-fold greater (Table 1).

Among all *P*. *aeruginosa* strains, 65% were susceptible to ciprofloxacin, 57.5% to levofloxacin, and 37.5% to moxifloxacin. Aside from one strain, all strains resistant to levofloxacin were also resistant to ciprofloxacin and moxifloxacin (n = 13). One strain was resistant to moxifloxacin (MIC = 3 μg/mL), but susceptible to ciprofloxacin (CMI = 0.19 μg/mL) and levofloxacin (MIC = 0.75 μg/mL). The MICs distributions were bimodal for each antibiotics tested (Fig 1B). Modal MICs and MICs_90_ of each antibiotic tested were all ≥32 μg/mL (Table 1).

Among all *S*. *maltophilia* strains, 15% were susceptible to ciprofloxacin, 35% to levofloxacin, and 52.5% to moxifloxacin. All strains intermediate or resistant to moxifloxacin were also resistant to the other quinolones tested (n = 19). Ciprofloxacin and levofloxacin MIC_50_ (4 and 1.5 μg/mL respectively) were higher than clinical lower breakpoint for susceptibility. Only moxifloxacin had a MIC_50_ (0.75 μg/mL) that was lower than the cut-off for resistance. MICs distribution was almost identical for each antibiotics tested (Fig 1C).

### Time kill studies at plasma peak concentrations

#### K. pneumoniae

**Fig 2:**
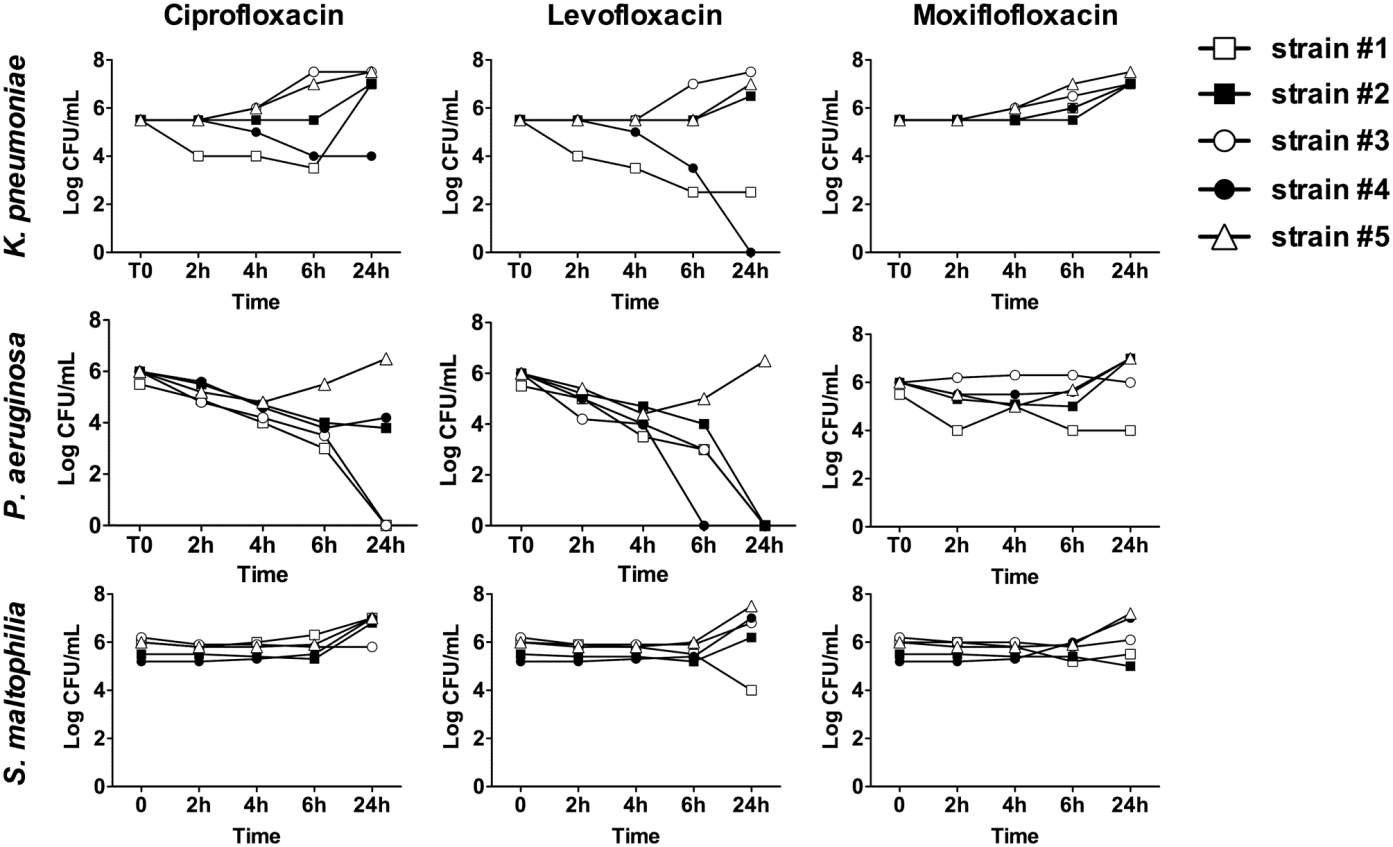
Time-kill studies with 5 strains of *K*. *pneumoniae*, *P*. *aeruginosa*, *S*. *maltophilia* with concentration equal to the theoretical plasma peak (ciprofloxacin = 4 μg/mL; levofloxacin = 10 μg/mL; moxifloxacin = 3 μg/mL).

Three strains among the 5 tested were susceptible to ciprofloxacin (strains 1, 3 and 4). Nevertheless, strains 1 and 4 had not have far lower MICs than strain 3 (50 and 15 times lower, respectively, Table 2). For these both strains, ciprofloxacin at peak concentration exerted a bactericidal activity, with a 2-log CFU/mL inoculum reduction in the first 6 h. A 2–6 h bacteriostatic activity was observed for the others strains. Then, regrowth occurred up to 24 h for all strains (Fig 2).

Four strains among the 5 tested were susceptible to levofloxacin (strain 1–4). Again, strains 1 and 4 had lower MICs than strains 2 and 3 (5 and 15 times lower, respectively, Table 2). A 4-5-log decrease was observed at 6 h for these strains. Bacteriostatic activity was observed for strain 1. A bacterial growth re-occurred at 24 h for strains 2 and 3 (resistant) (Fig 2).

Moxifloxacin exhibited only a 2–6 h bacteriostatic activity, whatever strains categorization, followed by regrowth up to 24 h. (Fig 2).

#### P. aeruginosa

Four strains among the 5 tested were susceptible to ciprofloxacin (strains 1–4) and one was intermediate (strain 5) (Table 2). At least a 24 h 5-log decrease occurred for strains 1 and 3, which are the most susceptible according to their MICs. A bactericidal activity, with 2.5-log inoculum reduction at 6 h, against strains 2 and 4 was observed. A bacterial growth re-occurred at 24 h for strain 5 (resistant) (Fig 2).

Two strains among five were susceptible to levofloxacin (strains 1 and 3) and three were intermediate (strains 2, 4 and 5) (Table 2). All strains except one were killed by levofloxacin at 5-log decrease level. Strain 5, which is resistant (MIC = 1.5 mg/L) was subject to a regrowth starting at the 4^th^ hour.

Two strains were susceptible to moxifloxacin (strains 1 and 3) and three were resistant to moxifloxacin (strains 2, 4 and 5) (Table 2). A bactericidal activity was observed for only one strain (strain 1), with a 2 log inoculum reduction at 24 h. For the others strains, whatever their MICs, a bacteriostatic activity was observed until 6 h, followed by regrowth (Fig 2).

#### S. maltophilia

No bactericidal activity was observed excepted for strain 1 with levofloxacin (Fig 2). All antibiotics exhibited a static effect to all strains at 6 h.

### Time-kill studies at one and two fold MICs

**Fig 3:**
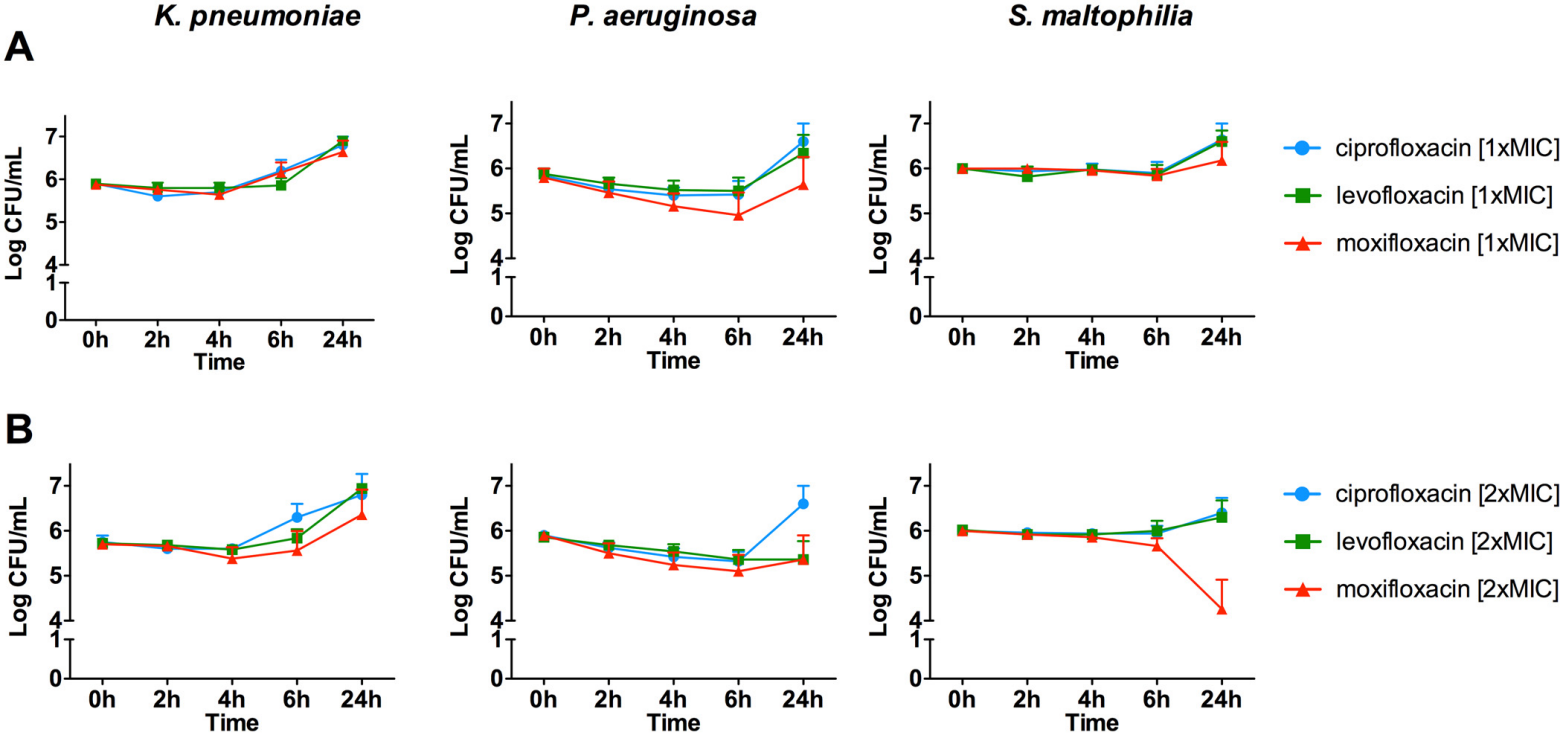
Time-kill studies with 5 strains of *K*. *pneumoniae*, *P*. *aeruginosa*, *S*. *maltophilia* with concentration equal to one fold (A) or two fold (B) the MIC of the strain against the considered antibiotic.

At concentrations equal to one (Fig 3A) or two fold (Fig 3B) MICs, the same profile was observed for all strains tested. A bacteriostatic activity was observed up to 6 h, followed by a regrowth at 24 h. No significant difference was observed between the antibiotics tested.

## Discussion

In a general manner, the bactericidal activity of ciprofloxacin and levofloxacin was inversely proportional to the MICs.

### K. pneumoniae

For *K*. *pneumonia* and fluoroquinolones, the break points are well separated from the ecoffs (epidemiological cut-off). In our population, for each antibiotic tested, MIC_50_ and modal MICs were similar, meaning that the majority of the strains were susceptible. In fact, our results clearly show two distinct sub-populations: a susceptible-one with MICs close to MIC_50_, and a resistant-one with MICs closer to MIC_90_. Interestingly our data seems to fit with EUCAST-ones. E-coff and modal MIC provided by EUCAST are 0.125 μg/mL and 0.03 μg/mL for ciprofloxacin, 0.25 μg/mL and 0.06 μg/mL for levofloxacin/moxifloxacin, respectively (EUCAST data). The ecoff of our *K*. *pneumoniae* population is about 0.38 μg/mL, indicating that our strains may be representative of general population. The MIC50 of the three antibiotics are very low and close one to the other (0,047, 0,094 and 0,064 μ g/mL, respectively), suggesting a similar *in vitro* activity against our susceptible strains. Thus, on a bacteriostatic point of view, the three antibiotics are equivalent on *K*. *pneumoniae*. We confirm this through time-kill curves at concentrations of each antibiotic of one or two fold the MICs resulting in a bacteriostatic activity. Nevertheless, in terms of bactericidal activity at higher concentration, equal to peak concentrations, it has been shown that the population density expressed in log-CFU is inversely proportioned to MICs values.

Nevertheless, levofloxacin seems to be somewhat more bactericidal than ciprofloxacin. It is not likely that this difference may have clinical relevance. The bactericidal activity of ciprofloxacin was high for both strains with low MIC, when it was much lower for. strain 3 the MIC of which (0.38 μg/mL; susceptible) is close to the clinical breakpoint (0.5 μg/mL). This should be a key point when using MICs. A MIC value close to clinical breakpoint could indicate that the antibiotic could be less effective than what can be expected when the MIC is far below from breakpoint. The same holds true with levofloxacin. Among the susceptible 4 strains, both strains with very low MICs undergo a deep bactericidal effect, whereas the two strains with MICs values closer to clinical breakpoint do not. Another limit of MICs is highlighted by the results obtained for moxifloxacin. The three antibiotics had very close and very low MIC_50_ values (0.047, 0.094 and 0.064 μg/mL respectively), suggesting an equivalent in *vitro* good activity against susceptible strains. But, on the contrary, time-kill studies indicated an absence of bactericidal activity against *K*. *pneumoniae*.

### P. aeruginosa

In our study, the modal MICs and MICs_90_ of our total population were identical (>32 mg/mL) for all molecules, showing that the majority of strains included were resistant to these antibiotics. In fact, our results show two distinct subpopulations: a susceptible one, the smallest, with modal MICs for ciprofloxacin, levofloxacin and moxifloxacin equal to 0.19, 0.38 and 1 to 1.5 μg/mL respectively and a larger resistant one. EUCAST e-coff and modal MIC are 0.5 μg/mL and 0.12 μg/mL for ciprofloxacin, 2 μg/mL and 0.5 μg/mL for levofloxacin, and 4 μg/mL and 1 μg/mL for moxifloxacin, respectively. In our study, the MICs_50_ in susceptible population are close to the modal MICs provided by EUCAST data. The lower clinical breakpoints for ciprofloxacin and levofloxacin are 1 μg/mL, being far higher from our modal MIC of the susceptible population. But moxifloxacin breakpoints at 1 μ g/mL is very close to modal MICs of our susceptible population and cover a part of wild strains. This correlates with EUCAST data, with an e-coff equal to 4 μg/mL, higher than clinical breakpoints.

In terms of bactericidal activity, at plasma peak concentration, ciprofloxacin and levofloxacin are equivalent on 2/5 strains tested (6-log reduction), levofloxacin is superior to ciprofloxacin on 2/4 strains tested (6-log *vs* 2-log reduction), and ciprofloxacin and levofloxacin have no bactericidal activity on 1/5 strain tested. As has been shown with *K*. *pneumoniae*, the lower the MIC the better the bactericidal activity. For ciprofloxacin at 24 h, the best (6 log-decrease) killing activity was observed for both strains with the lower MICs. The same occurs for levofloxacin, with a 6-log decrease for strain 1, 2 and 4. Ciprofloxacin, as already shown in the literature, seems to have a good efficiency against susceptible *P*. *aeruginosa* strains [18–21]. However, levofloxacin seems to have a better activity against *P*. *aeruginosa* than ciprofloxacin. For the five strains tested by time-kill studies, a bactericidal effect of levofloxacin was observed at 6 h, although some of these strains (strain 2, 4 and 5) were intermediate to levofloxacin, and a total bacterial killing was observed at 24 h for 4/5 strains. It is not likely that this difference may have clinical relevance. Gillespie *et al*. have shown that levofloxacin less frequently favours resistant mutants appearance than ciprofloxacin, but no difference between the two molecules was observed in their time-kill studies [18]. The few number of tested strains by our time kill studies (n = 5) does not allow us to affirm a real superiority of levofloxacin on ciprofloxacin against *P*. *aeruginosa*.

### S. maltophilia

In our study, levofloxacin and moxifloxacin MICs_50_ and modal MICs are significantly lower than those of ciprofloxacin. Moxifloxacin MIC_90_ was significantly lower than levofloxacin and ciprofloxacin. Moxifloxacin seems therefore to have a better *in vitro* activity. EUCAST does not provide e-coff for *S*. *maltophilia* and quinolones. The only available data are modal MICs: ciprofloxacin 2 μg/mL, 1 μg/mL for levofloxacin and 0.25 μg/mL for moxifloxacin (EUCAST data). In comparison, our strains are more resistant to ciprofloxacin but have the same profile for levofloxacin and moxifloxacin as worldwide strains. For those three antibiotics, clinical breakpoints are very close to modal MICs of population and cover a part of wild strains.

Time kill studies show a little activity of fluoroquinolones against *S*. *maltophilia*. A bactericidal activity was only observed for one strain (2-log reduction) with levofloxacin. For the other strains, a bacteriostatic activity, followed or not by regrowth was observed.

## Conclusion

In our study, the bactericidal activity has been shown to be inversely proportioned to MICs values. A “susceptible” MIC value close to clinical breakpoint could indicate that the antibiotic could be less effective than what can be expected from a strain with a much lower MIC far from the clinical breakpoint. Our data go in the same meaning as *Torres* et al. who have recently shown that “highly susceptible” isolates are associated with higher clinical cure rates than “borderline isolates”[22].This consideration should be taken into account when choosing between different antibiotic that are all “susceptible”.
